# Pan-Genome Analysis of *Laribacter hongkongensis*: Virulence Gene Profiles, Carbohydrate-Active Enzyme Prediction, and Antimicrobial Resistance Characterization

**DOI:** 10.3389/fmicb.2022.862776

**Published:** 2022-03-31

**Authors:** Pei-Bo Yuan, Yi Zhan, Jia-Hui Zhu, Jia-Hui Ling, En-Zhong Chen, Wan-Ting Liu, Lin-Jing Wang, Yu-Xia Zhong, Ding-Qiang Chen

**Affiliations:** Department of Laboratory Medicine, Microbiome Medicine Center, Zhujiang Hospital, Southern Medical University, Guangzhou, China

**Keywords:** pan-genome analysis, *L. hongkongensis*, virulence gene, carbohydrate-active enzyme, antimicrobial resistance

## Abstract

*Laribacter hongkongensis* is a new emerging foodborne pathogen that causes community-acquired gastroenteritis and traveler’s diarrhea. However, the genetic features of *L. hongkongensis* have not yet been properly understood. A total of 45 aquatic animal-associated *L. hongkongensis* strains isolated from intestinal specimens of frogs and grass carps were subjected to whole-genome sequencing (WGS), along with the genome data of 4 reported human clinical strains, the analysis of virulence genes, carbohydrate-active enzymes, and antimicrobial resistance (AMR) determinants were carried out for comprehensively understanding of this new foodborne pathogen. Human clinical strains were genetically more related to some strains from frogs inferred from phylogenetic trees. The distribution of virulence genes and carbohydrate-active enzymes exhibited different patterns among strains of different sources, reflecting their adaption to different host environments and indicating different potentials to infect humans. Thirty-two AMR genes were detected, susceptibility to 18 clinical used antibiotics including aminoglycoside, chloramphenicol, trimethoprim, and sulfa was checked to evaluate the availability of clinical medicines. Resistance to Rifampicin, Cefazolin, ceftazidime, Ampicillin, and ceftriaxone is prevalent in most strains, resistance to tetracycline, trimethoprim-sulfamethoxazole, ciprofloxacin, and levofloxacin are aggregated in nearly half of frog-derived strains, suggesting that drug resistance of frog-derived strains is more serious, and clinical treatment for *L. hongkongensis* infection should be more cautious.

## Introduction

*Laribacter hongkongensis* (*L. hongkongensis*) is a gram-negative foodborne organism. It is firstly discovered in the stool of six patients with diarrhea in Hong Kong and then reported to be associated with community-acquired gastroenteritis and stomach and intestinal infection or traveler’s diarrhea (Yuen et al., 2001; Woo et al., 2004), and is suggested to be distributed around the world according to the global case reports (Teng et al., 2005; Woo et al., 2005; Kim et al., 2011; Beilfuss et al., 2015; Engsbro et al., 2018; Hung et al., 2020). *L. hongkongensis* has been also discovered in the gut of diverse aquatic animals, birds, drinking water reservoirs, aquatic environments, as well as wastewaters (Lau et al., 2007a,b, 2009; Ni et al., 2011; Greay et al., 2019). Frog and freshwater fish have been recognized to be the primary hosts for *L. hongkongensis* and the sources of human infection. Eating raw fish, as well as inadequate food cleaning and disinfection, can lead to an increase in foodborne *L. hongkongensis* infections.

Since *L. hongkongensis* was identified and confirmed as pathogenic by Koch’s Law (Beilfuss et al., 2015), a series of studies have been conducted to understand its pathogenicity and epidemiology. The serological detection methods were established (Tse et al., 2014; Wang et al., 2017). The adaptive response and transcriptional regulation patterns of *L. hongkongensis* coupled with different nutritional sources, aerobic or anaerobic conditions, and different temperatures were also studied (Xiong L. et al., 2015; Kong et al., 2016, 2017; Xiong et al., 2017, 2019). Researchers also discussed the pathogenicity factors of *L. hongkongensis* (Xie et al., 2014). Drug-resistant properties and the presence of clinical multiple drug-resistant strains have also been reported (Raja and Ghosh, 2014; Wu et al., 2018; Teng et al., 2021). However, these studies were conducted on specific isolated strains. And all 4 genomes published were isolated from clinical human samples. To better understand the genetic and pathogenic features of *L. hongkongensis*, and to assess the risk of infection from the main primary hosts of *L. hongkongensis*, a large-scale genome-based analysis involving strains from Frogs and freshwater fishes should be carried out.

Here, we conducted a pan-genome analysis of 45 aquatic frogs and grass carps isolated strains and all 4 clinically reported strains with genome data. A comprehensive analysis of the genetic evolution, virulence factors, carbohydrate-active enzymes, drug-resistant genes, and phenotypes were performed to assess the influence of bacteria’s source and lineage on pathogenicity risk, providing a reference for further studies on source tracing and treatment of *L. hongkongensis* related infections.

## Materials and Methods

### Bacterial Isolation

Intestinal specimens of grass carp and frog were collected from retail markets in Guangzhou. Through isolation, culture, biochemical identification, 45 strains were identified as *L. hongkongensis.* Among the 45 strains, 21 strains were isolated from grass carps, and 24 strains from frogs. Strains isolated from frog were named with an initial letter W (indicating WA in Chinese), and strains isolated from grass carp were named with an initial letter Y (indicating YU in Chinese).

### Preparation of Genomic DNA and Whole-Genome Sequencing

Genomic DNA was extracted from freshly grown cells by a bacterial DNA extraction kit (Dongsheng Biotech, CHN). The genomic DNA was mechanically sheared using a Nebulizer instrument (Invitrogen) to select fragments of approximately 550 bp. A DNA library was prepared using the Illumina TruSeq™ Nano method and sequenced on the Illumina MiSeq platform with the 2 × 250 bp paired-end (PE) reagent kit v2. The quality of the raw reads was checked in Fast QC v0.11.5,^1^ and low-quality reads were filtered. Reads with adapter, ≥ 10% unknown bases, or > 40% low-quality bases (Q ≤ 10) were designated as low-quality reads. Filtered clean reads were assembled into contigs with edena v3 by default parameters (Hernandez et al., 2014), which is based on the classical graph approach and known for its efficiency in handling base errors and detecting potentially spurious reads. The genome assembly quality was further evaluated by quast v5.0.2 (Gurevich et al., 2013).

The genome sequences and associated data for 45 new sequenced *L. hongkongensis* strains reported in this study were deposited in NCBI under the BioProject accession number PRJNA770832.

### Genome Preparation, Annotation, and Pan-Genome Inference

Four *L. hongkongensis* genome sequences of human clinical strains were downloaded from NCBI.^2^ All 49 *L. hongkongensis* genomes were annotated *de novo* with Prokka (Seemann, 2014) version 1.14.5. Function annotation and orthology prediction of all genes was conducted using eggNOG 5.0 (Huerta-Cepas et al., 2019). The Prokka produced GFF3 format annotation files were provided to the rapid large-scale prokaryote pan-genome analysis tool Roary version 3.12.0 (Page et al., 2015) to calculate the pan-genome of *L. hongkongensis*. Coding regions were extracted from the input and converted to protein sequences, filtered to remove partial sequences, and iteratively pre-clustered with CD-HIT, then an all-against-all comparison was performed with a built-in BLASTP on the reduced sequences with the default sequence identity cutoff of 95%. Roary produced a gene presence/absence matrix, a multi-FASTA alignment of core genes using PRANK (31) version 170427, and determined the subsets genes of core and pan-genomes. The gene presence/absence matrix was visualized using the built-in heatmap function of R with presence shown in red and absence in blue. The strains were clustered by row-side dendrogram generated with the default complete linkage clustering method using the euclidean distance measure.

For the statistical extrapolation, non-linear least squares curve fittings of the observed core and pan genome sizes as functions of the number of analyzed genomes were performed with GraphPad Prism 8 following the models/regression algorithms given by researchers (Tettelin et al., 2005, 2008; Nourdin-Galindo et al., 2017). Curve fitting the pan-genome was performed using a power-law regression based on Heaps’ law [*y* = *A*_*pan*_*x^B_pan_^*], as previously described (Tettelin et al., 2008; Nourdin-Galindo et al., 2017), where y was the pan-genome size, x was the number of analyzed genomes, A_pan_ and B_pan_ were the fitting parameters. B_pan_ was equivalent to the γ parameter in estimating an open or closed pan-genome (Tettelin et al., 2008). When 0 < B_pan_ < 1 indicates an open pan-genome, while B_pan_ < 0 or B_pan_ > 1 indicate a closed pan-genome. Curve fitting of the core-genome was performed using an exponential decay regression model [*y* = *A*_*core*_*e*^(−*B*_*core*_*x*)^*C*_*core*_], where y was the core-genome size, x was the number of analyzed genomes, C_core_ was the extrapolated size of the core genome for x→∞, A_core_ and B_core_ were the fitting parameters. Curve fittings of new genes discovered with the availability of additional genome sequences were performed using both a power-law regression based on Heaps’ law [*y* = *A*_*new*_*x*^−*B*_*new*_^], and an exponential decay regression model [*y* = *C*_*new*_*e*^(−*D*_*new*_*x*)^*E*_*new*_], where y was the new gene number, x was the number of analyzed genomes, and A_new_, B_new_, C_new_, D_new_, and E_new_ were the fitting parameters. The B_new_ was equivalent to the α parameter in estimating an open or closed pan-genome (α ≤ 1, the pan-genome is open; α > 1, the pan-genome is closed), while E_new_ was equivalent to the tg(θ) parameter representing the number of new genes asymptotically predicted for further genome sequencing (Tettelin et al., 2008).

### Phylogenetic Analyses Based on Core Genome

A phylogenetic tree of 49 *L. hongkongensis* strains was built based on protein sequences of core genes with the PhyloPhlAn pipeline v3.0.60 (Asnicar et al., 2020). Core genes were determined by Roary, and their corresponding protein sequences from HKU1 were extracted as markers. Amino acid sequences predicted by prokka for core genes were extracted from genomes as inputs. The programs FastTree and RAxML (Stamatakis, 2014) were used to build the trees using the PhyloPhlAn 3.0.60 database in an accurate mode with the diversity level set as low. Specifically, homologs were first identified and extracted using Diamond 0.9.21 (Buchfink et al., 2021) with the following command “blastp –more-sensitive –id 50 –max-hsps 35 -k 0.” Each variant of each marker was then aligned using MAFFT 7.487 (Katoh and Standley, 2013), with command “–anysymbol –auto.” The generated multiple-sequence alignments were concatenated and trimmed using trimAl 1.4.rev15 (Capella-Gutiérrez et al., 2009), with “-gappyout” option. A maximum likelihood phylogenetic tree was produced with LG-CAT model using FastTree 2.1.10 (Price et al., 2010), with command “-quiet -pseudo -spr 4 -mlacc 2 -slownni -fastest -no2nd -mlnni 4 –lg.” The phylogeny was then refined using RAxML 8.2.12 (Stamatakis, 2014), with “-p 1989 -m PROTCATLG” options. The tree was rooted by the mid-point method, and visualized and edited online at the website of iTOL (Letunic and Bork, 2021).

### Virulence Factors Identification

Prediction of virulence genes was performed through searches querying each genome against the Virulence Factor Database (Liu et al., 2019) with the VFanalyzer web server. All parameters used were set as default. Based on sequences of Virulence Factor, another phylogenetic tree of 49 *L. hongkongensis* strains was built with Parsnp 1.5.6 (Treangen et al., 2014), then rooted by the mid-point method and visualized with iTOL v5 (Letunic and Bork, 2021).

### Carbohydrate-Active enZYme Annotation

The fasta file of 49 *L. hongkongensis* strains was subjected to automated annotation and assignment to CAZymes using the dbCAN2 meta server (Zhang et al., 2018). Three tools, HMMER (for annotated CAZyme domain boundaries according to the dbCAN CAZyme domain HMM database), DIAMOND (for fast blast hits in the CAZy database), and Hoptep (for short conserved motifs in the PPR library) were used with the default *E*-value and coverage cutoff. All descriptions and classifications were compiled from CAZy (Lombard et al., 2014).

### Antimicrobial Resistance Determinants Identification and Antimicrobial Susceptibility Testing

Prediction of antibiotic resistance genes was performed using RGI (v.3.1.1) (Alcock et al., 2020). All isolates were tested for antimicrobial susceptibility by the disk diffusion method using Mueller-Hinton agar and commercially available discs (Oxoid). The antimicrobial agents used were Amikacin (30 μg), Ampicillin (10 μg), Ampicillin-Sulbactam (30 μg), Aztreonam (30 μg), Cefazolin (30 μg), Cefepime (10 μg), Ceftazidime (30 μg), Ceftriaxone (30 μg), Cefuroxime (30 μg), Ciprofloxacin (5 μg), Gentamicin (10 μg), Imipenem (10 μg), Levofloxacin (5 μg), Meropenem (10 μg), Minocycline (30 μg), Rifampicin (5 μg), Tetracycline (30 μg), Trimethoprim-Sulfamethoxazole (25 μg). *Escherichia coli* ATCC25922 is used as a quality control organism. The Antibiotic susceptibility was interpreted based on Clinical and Laboratory Standards Institution (CLSI) guidelines.

## Results and Discussion

### Pan-Genome Overview

**FIGURE 1:**
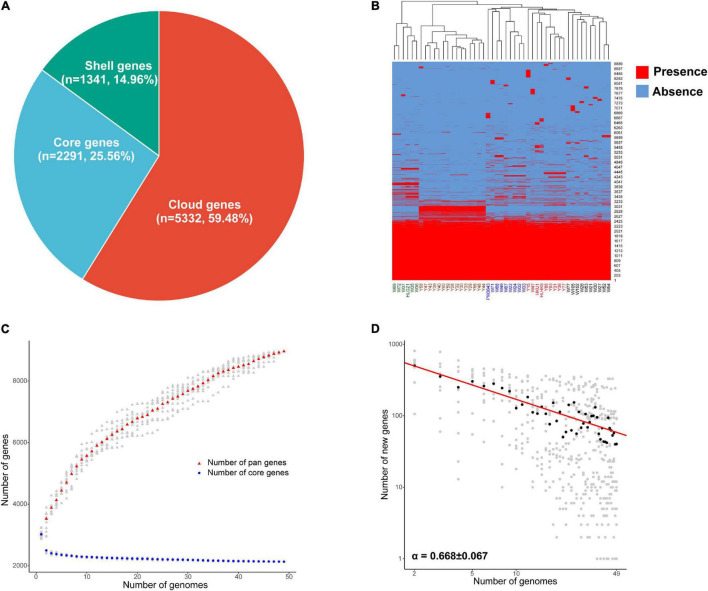
Pan-genome analysis of 49 *L. hongkongensis* strains. **(A)** Composition of core, shell, and cloud genes in pan-genome. **(B)** Presence and absence of all identified genes. Presence was shown in red and absence was shown in blue, the clustering of samples based on genes’ presence and absence was displayed above the heatmap. **(C)** The core and pan-genome development plots. The number of pan genes was shown by red dots, and core genes by blue dots, with *L. hongkongensis* genome sizes shown on the horizontal axis. **(D)** The log-log plot of Power law regression for new genes discovered with the availability of additional genome sequences. Medians of the distributions were indicated by black dots, the exponent α was from the Heaps’ law *n* = *kN*^−α^, where n was the new gene number, N was the number of analyzed genomes, α can be used to estimating an open or closed pan-genome (α ≤ 1, the pan-genome is open; α > 1, the pan-genome is closed). Strains isolated from frog were named with an initial letter W, and strains isolated from grass carp were named with an initial letter Y, human clinical strains were presented in strong pink, strains named HKU1, HLHK9, and PW3643 were isolated from Hong Kong patients, while HLGZ1 was isolated from a Guangzhou patient.

The average full genome size and GC content of the 49 *L. hongkongensis* strains were 3.19 Mb and 62.5%, with an average of 2,997 coding sequences, respectively. The general characteristics of *L. hongkongensis* genomes can be found in Supplementary Table 1. The pan-genome of *L. hongkongensis* was inferred with Roary. Roary produced a total of 8,964 protein-coding gene sequence clusters. The “core genome,” consisting of genes present in at least 95% of strains (≥47 out of 49), was represented by 2,291 genes (≈26% of all genes). The 6,673 non-core genes were divided into 1,341 “shell genes” (i.e., shell genes present in at least 15% of strains, and no more than 95% of strains; ≈15% of all genes), and 5,332 “cloud genes” (i.e., cloud genes present in no more than 15% strains, ≤ 7 out of 49 strains; ≈59% of all genes) (Figure 1A). A heatmap was drawn to visualize the presence/absence of all 8,964 genes in 49 genomes (Figure 1B). The strains can be divided into 6 clusters for holding specific genes. Interestingly, there were 203 genes only held by some strains from grass carps (the second cluster in brown word label). Though most of these genes can only be annotated as function poorly characterized, some genes were annotated to be involved with information storage and processing, indicating they might be important for their adaptation of intestinal specimens of grass carps. The detailed gene presence and absence matrix and annotation of all genes can be found in Supplementary Table 2.

To determine whether the pan-genome was open or closed, the core and pan-genome development plots of *L. hongkongensis* were drawn as Figure 1C, the number of genes in the pan-genome increased as more genomes were sequenced, whereas the number of genes in the core-genome decreased. The pan-genome of *L. hongkongensis* could be considered “open,” as supported by the growth exponent value of 0.3019 ± 0.005 (95% confidence interval), based on B_pan_ (equivalent to γ in the reference research) parameter from Heaps’ law. The exponential decay regression fitting of core-genome revealed an extrapolated core genome size of 2,198 (95% confidence interval 2,192–2,205), based on the value of the C_core_ parameter. Furthermore, the power law and exponential regression fits for new genes discovered with the availability of additional genome sequences were performed, and the log-log plot was built (Figure 1D). The B_new_ (equivalent to α in the reference research) parameter of power law (B_new_ = 0.668 ± 0.066 < 1) was in good agreement with the value of B_pan_ (0 < B_pan_ = 0.3019 < 1), indicating the openness of the pan-genome. The value of parameter E_new_ [tg(θ)] in exponential regression fit indicated that each additional genome sequence would add asymptotic 71.24 ± 14.91 (95% confidence interval) new genes to the pan-genome. The open pan-genome suggested the ability of *L. hongkongensis* to adapt to new niches by generating or incorporating new genes, which might support its virulence and host adaption.

### Function Annotation and Phylogenetic Analysis of Core Genes

**FIGURE 2:**
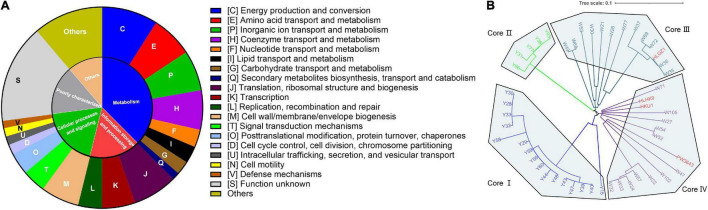
Function annotation and phylogenetic tree of *L. hongkongensis* core genes. **(A)** COG Function categories of core genes. The annotation was conducted with eggNOG based on the COG database. **(B)** Phylogenetic tree of 49 *L. hongkongensis* strains based on amino acid sequences of core genes inferred with PhyloPhlAn 3.0.60. The genes can be grouped into eggNOG orthologous groups, but cannot be grouped into any COG function categories by eggNOG were labeled as “others.”

The functions of 2,291 core genes were characterized by matching the sequences with the COG database, giving out the conserved functions which may play housekeeping roles in *L. hongkongensis* (Figure 2A and Supplementary Table 3). The core genes were annotated in metabolism (37.54%), information storage and processing (16.32%), cellular processes and signaling (19.03%), and about 28% of core genes are functions unknown. It is worth mentioning that numerous core genes are annotated in processes like signal transduction mechanisms (87 genes), intracellular trafficking, secretion, and vesicular transport (32 genes), defense mechanism (27 genes), indicating that these processes may be important for the survival of *L. hongkongensis* in the various living environments and may play key roles in interaction with host immune systems.

A phylogenetic tree of 49 *L. hongkongensis* strains was constructed based on amino acid sequences of 2,291 core genes to understand the genetic relationship between strains from different sources (Figure 2B). The strains can be divided into four clusters in the tree. The strains in the core I cluster and core II cluster were derived from the grass carp samples, while strains in the core III and core IV were mostly derived from the frog samples except for HLGZ1, HKU1, and HLHK9, which were collected from human clinical samples. The results of the phylogenetic tree suggested that evolutionary divergence between strains of different hosts had occurred in core genes. Different *L. hongkongensis* strains might have their host preference.

For the clinical strains, HKU1 and HLHK9 were closely related with each other, along with PW3643, they were more related with strains derived from frogs, like W71, W105, and W47 in the core IV cluster. HLGZ1 was closely related with W36 and W35 in the core III cluster, indicating that human clinical strains were genetically more related to some strains from frogs. HLGZ1 was collected by a diarrheic human stool sample and reported to be clustered into the same MLST cluster with two frog isolates (Wu et al., 2018). These results were consistent with the hypothesis that some *L. hongkongensis* strains derived from frogs may have pathogenicity potential for humans (Wang et al., 2019).

### Virulence Factor Profiles

To further understand pathogenic feathers and measure the disease risk of these *L. hongkongensis* food-associated isolates, we analyzed the virulence factors in each isolate. There were 112 virulence factors detected in total, with an average of about 65 virulence factors among the 49 *L. hongkongensis* strains (Supplementary Table 4). They were contributing to invasion, adherence, immune evasion, efflux pump, toxin, motility, stress adaption, and other virulence-related functions of *L. hongkongensis*.

**FIGURE 3:**
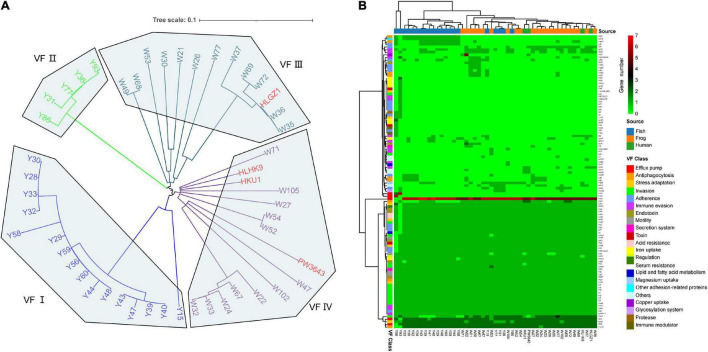
Virulence factor profiles of 49 *L. hongkongensis* genomes. **(A)** Phylogenetic tree based on amino acid sequences of virulence factors using the Maximum Likelihood Method by parsnp. Human clinical strains were presented in strong pink. **(B)** Complex heatmap of predicted virulence genes in 49 genomes. Clustering was performed on row and column distances by ward. D2 method. The gene copy numbers, sample sources, and virulence factor classes were present indicated with different colors.

Another phylogenetic tree of 49 *L. hongkongensis* strains was constructed based on sequences of virulence genes (Figure 3A). The strains were divided into 4 clusters same as the tree based on core genes, indicating that the evolution of virulence genes was coordinated with the evolution of the core genome in *L. hongkongensis*. Virulence gene variation might play important role in bacterial adaptation to the host environment.

To further understand the virulence-related difference between different sources, a heatmap was drawn to visualize the distribution of the virulence genes (Figure 3B). Forty-eight core virulence factors were present in more than 47 strains, and 22 specified virulence factors were only present in one single strain. Strain Y93 and Y56 were distinguished from all other strains for holding 12 and 5 specified virulence factors and with the absence of 14 and 11 core virulence factors. Based on the clustering of samples, clinical strain HLGZ1 and HLHK9 were clustered with W36 and W37, PW3643 and HKU1 were clustered with each other, along with W27, W32, W24, and W33, indicating human clinical strains were more similar with strains isolated from frogs on virulence factors. This also suggested that strains isolated from frogs may have a higher potential to infect humans, which adds strength to the previous viewpoint (Wang et al., 2019). There were no critical specified VF genes shared by all 4 clinical derived strains, though *gmd* [LPS(Brucella), 3/4 VS 7/49, held by HKU1, HLGZ1, and PW3643], *ddhC* (O-antigen (Yersinia), 2/4 VS 3/49, held by HKU1 and PW3643), and *wcaG* (O-antigen (Yersinia), 2/4 VS 3/49, held by HKU1 and PW3643) were enriched in these human clinical strains, indicating these genes may serve important roles for human infection of *L. hongkongensis*.

Three virulence factors were noticed to be specific of stains from grass carps, *orfL* (adherence), *algC*(Antiphagocytosis), and *pdhB* (other adhesion-related proteins), existing in more than 50% of grass carp derived strains. *AlgC* encodes a phosphoglucomutase, which is essential for biofilm production, and also involved with the formation of protective barrier against host immune defenses and antibiotics and might be important for *L. hongkongensis* infection and survival in grass carps. There was also one gene *wecC* that only existed in grass carp (52.4%) or frog (17.4%) derived strains, and have not been shown in clinical strains, it was known to be involved in the pathway enterobacterial common antigen biosynthesis and in bacterial outer membrane biogenesis (Meier-Dieter et al., 1990). Two genes, *flpF*, and *gmd* were only existed in human (50%) or frog (73.9%) derived strains, and have not shown in grass carp derived strains. One virulence gene, *algA* was found in all strains from frog and human, but only in 47.6% of strains from grass carp, it is known to produce a precursor for alginate polymerization (Shinabarger et al., 1991). The alginate layer provides a protective barrier against host immune defenses and antibiotics and might contribute to survival in the host. The specific virulence-related genes held by strains from different sources might indicate that different strategies have been adopted for multi-host bacteria to adapt to drastic environmental changes.

### Carbohydrate-Active enZYme Profiling

**FIGURE 4:**
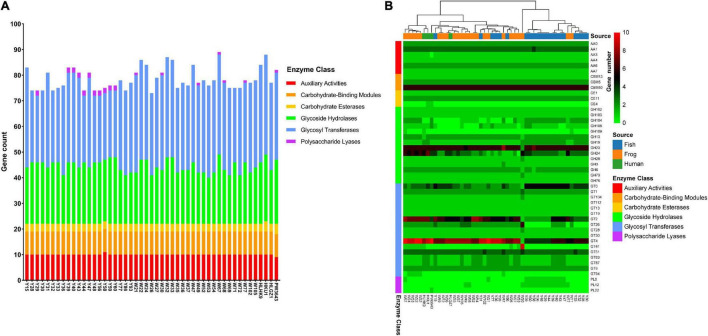
Carbohydrate-Active Enzyme (CAZy) profiles of 49 *L. hongkongensis* genomes. **(A)** Stacked bar chart of Carbohydrate-Active Enzymes distributions. **(B)** Complex heatmap based on gene copy numbers of Carbohydrate-Active Enzymes in each genome. Clustering is only performed on row distance by ward. D2 method. Analysis of the CAZy profiles was annotated using the dbCAN2 resource CAZy family-specific hidden Markov models (HMMs) (Zhang et al., 2018). The sources of strains and the classes of CAZyme modules or domains were represented as colored bars.

Carbohydrate utilization is the core process for organism survival and reflects the adaptability of bacterial strains to environments, and provides hints for understanding the interaction between bacteria and host. CAZyme profiling was analyzed using dbCAN2 to investigate the genomic potential for carbohydrate utilization. The *L. hongkongensis* strains generally had similar types of CAZymes, but there were variations in the absolute numbers of genes within each of certain categories in the CAZy profiles (Figure 4A and Supplementary Table 5). The mean value of carbohydrate utilization gene number in 49 strains was 78.6. The gene numbers of Auxiliary Activities, Carbohydrate-Binding Modules, Carbohydrate Esterases, and Glycoside Hydrolases were similar between strains from different sources, but strains isolated from frogs and humans carried significantly more genes of Glycosyl Transferases than strains isolated from grass carps (*t*-test *p* = 1.49e^–5^).

A heatmap was drawn to visualize the presence and absence of Carbohydrate-active enzymes among 49 strains (Figure 4B). Eight gene families were present in almost all 49 strains with average gene numbers bigger than 3, indicating their important roles for *L. hongkongensis*. These gene families were AA1 (Levasseur et al., 2013), CBM50 (Boraston et al., 2004), GH23, GH24 (Henrissat and Davies, 1997), GT0, GT2, GT4, GT51 (Coutinho et al., 2003), which were reported to be multicopper oxidases that use diphenols, enzymes cleaving either chitin or peptidoglycan, lysozymes, cellulose synthase, sucrose synthase, and murein polymerase, and so on.

Human clinical strains seem to have more enzymes of GT4 compared to other strains, the mean value of GT4 enzyme number existed in 4 clinical strains was 8.75, while it was 7.7 in frog-derived strains, and 6.0 in grass carp derived strains. GT4 is the Glycosyltransferase family that contains sucrose synthase, sucrose-phosphate, and other Glycosyltransferases (Campbell et al., 1997), which may be important for *L. hongkongensis* survival inside the human host. PW3643 was the only strain that encoded PL22. PL22s were commonly referred to as oligogalacturonide lyases (OGLs). This enzyme family was found primarily in phytopathogenic or intestinal bacteria where it played a role in the metabolism of pectin (Abbott et al., 2010). PW3643 was also the only strain that do not have an AA3. Enzymes of family AA3 were widespread and catalyzed the oxidation of alcohols or carbohydrates with the concomitant formation of hydrogen peroxide or hydroquinones, the enzymes were most abundant in fungi and only recently found in bacteria (Sützl et al., 2018). CE4 was only found present in PW3643 and HKU1. Known activities of CE 4 family members include acetylxylan esterases, chitin deacetylases, chitooligosaccharide deacetylases, and peptidoglycan deacetylases (Hayhurst et al., 2008). Peptidoglycan was the essential bacterial cell wall polymer, indicating that PW3643 and HKU1 may have specified feathers in their cell wall. Interestingly, we found that clinical strains from Hong Kong, HKU1, and HLHK9 were clustered together with frog-derived strains, strain HLGZ1 was still clustered with W36 and W37 based on CAZyme profiling, consistent with the phylogenetic trees and virulence factors.

We also noticed that only some strains isolated from grass carp uniquely held genes of Polysaccharide Lyases PL0 and PL12, this may be due to the specified polyanionic substrates they need to degrade in their living habitat.

### Antimicrobial Resistance Determinants

**FIGURE 5:**
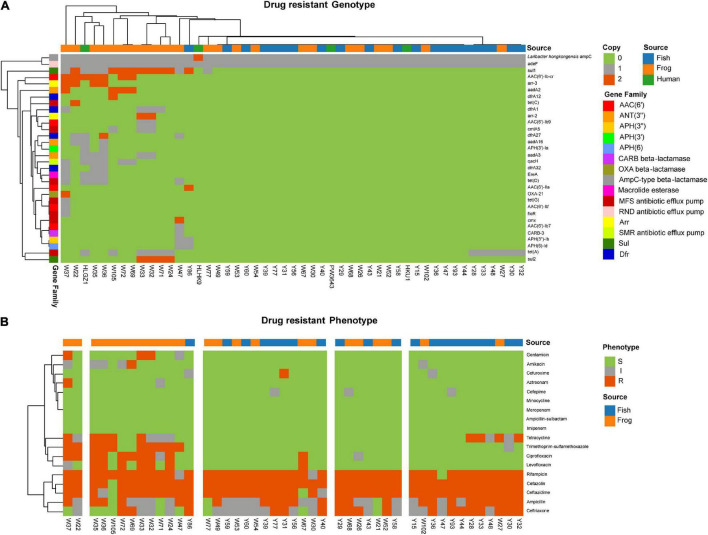
Antibiotic susceptibility and genetic determinants for *L. hongkongensis* strains in this study. **(A)** Genetic determinants of 49 *L. hongkongensis* were displayed in a Complex heatmap. The source of strains, gene copies, and gene families are represented as colored bars. **(B)** Antibiotic susceptibility of 45 aquatic animal isolated strains was displayed, with S indicating susceptible, I indicating intermediate, and R indicating resistance.

Antimicrobial resistance is the core focus of clinical treatment and control of pathogenic microorganisms. So, the antibiotic resistance genes were predicted and antimicrobial susceptibility for common drugs were tested. Thirty-two AMR genes or point mutations were identified (Figure 5A and Supplementary Table 6). Among all detected genes or point mutations, the *L. hongkongensis ampC* which encoded an class C beta-lactamase, and *adeF* which encoded resistance-nodulation-cell division (RND) antibiotic efflux pump had much higher prevalence rates than the other genes or point mutations (both 100%), followed by sulfonamide resistant *sul1* (30.6%), major facilitator superfamily (MFS) antibiotic efflux pump [24.5% *tet(A)*, 10.2% *tet(D)*, 2.0% *tet(C)*, and 2.0% *tet(G)*], aminoglycoside resistant gene *AAC(6′)*(14.3% *AAC(6′)-Ib-cr*, 4.1% *AAC(6′)-Ib9*, 2.0% *AAC(6′)-IIa*, 2.0% *AAC(6′)-Ib′*, and 2.0% *AAC(6′)-Ib7*), small multidrug resistance (SMR) antibiotic efflux pump *qacH* (12.2%), genes that encode proteins for streptomycin nucleotidylylation *ANT(3″)* (10.2% *aadA3*, 8.2% *aadA2*, and 6.1% a*adA16*), genes that encode proteins for streptomycin phosphorylation [4.1% *APH(3″)-Ia*, 4.1% *APH(3″)-Ib*, and 4.1% *APH(6)-Id*], trimethoprim resistant dihydrofolate reductase *dfr* (8.2% *dfrA27*, 8.2% *dfrA1*, and 6.1% *dfrA32*, and 2% *dfrA12*), rifampin ADP-ribosyltransferase (8.2% *arr-3*, 4.1% *arr-2*), macrolide esterase *EreA* (8.2%), genes encoding phenicol antibiotic efflux pump (6.1% *cmlA5*, and 2% *cmx*, and 2% *floR*), and OXA beta-lactamase *OXA-21* (2%), CARB beta-lactamase *CARB-3* (2%).

Susceptibility to 18 clinical commonly used antibiotics was tested for the 45 food-associated strains (Figure 5B). *L. hongkongensis* had a general resistance to cefazolin [97.8% Resistance (R) or intermediate (I)], rifampicin (97.8% R or I), ceftazidime (95.6% R or I), ampicillin (91.1% R or I), and ceftriaxone (80.0% R or I), this may be due to the high prevalence rate of *Laribacter hongkongensis ampC*. One-third of strains showed resistance to tetracycline due to *tet(A)* and other *tet* genes. Resistance/intermediate rate of frog-derived strains to fluoroquinolones (Ciprofloxacin, *p* = 5.45e^–5^, Levofloxacin, *p* = 2.83e^–3^), aminoglycosides (Amikacin, *p* = 1.32e^–2^, Gentamicin, *p* = 5.15e^–2^), Ceftriaxone (*p* = 1.20e^–3^), and Trimethoprim-sulfamethoxazole (*p* = 3.32e^–3^) were significantly higher compared to grass carp-derived strains. Among frog-derived strains, 50% of strains were resistant to ciprofloxacin, 33.3% of strains were resistant to levofloxacin, 41.7% of strains were resistant to trimethoprim-sulfamethoxazole, 25% of strains were resistant to amikacin, and 16.7% of strains were resistant to gentamicin. It was also worth mentioning that the resistance/intermediate rate of cefuroxime was higher (*p* = 5.71e^–2^) in grass carp derived strains (14.3%, 3/21) than in frog-derived strains (0%), which cannot be clearly explained by their AMR gene, this may due to unknown genomic differences in grass carp-derived strains.

The drug resistance of *L. hongkongensis* from frogs and fishes has been reported (Feng et al., 2012; Wang et al., 2019). We believe that the high resistance rates of frog-derived strains to fluoroquinolones and aminoglycosides might be due to prophylactic use of antibiotics in aquaculture and antibiotic pollution in aquaculture ponds, leading to selection pressure on environmental bacteria to develop resistance and to facilitate horizontal gene transfer (HGT) between bacteria. Sulfametoxydiazine, sulfamethazine, sulfamethoxazole, oxytetracycline, chlortetracycline, doxycycline, ciprofloxacin, norfloxacin, and enrofloxacin had been detected in sediment and water samples in Guangdong province, China (Xiong W. et al., 2015). Fluoroquinolones (Turnipseed et al., 2012) and multidrug-resistant bacteria (Lei et al., 2019) had also been detected from frog tissue. One research had reported that ARG pollution was more serious in bullfrog ponds than polyculture ponds due to the more frequent use of antibiotics in bullfrog rearing operations (Yuan et al., 2019).

The general beta-lactamase resistance of *L. hongkongensis* should be noted and clinical medication strategies adjusted accordingly. The high drug resistance rate of frog-derived strains is becoming a serious problem. We also noticed that drug-resistant frog-derived strains like W35, W36, W37, and so on were closely related with human clinical strains based on phylogenetic trees, virulence factors, and carbohydrate-active enzymes, indicating their high risk of human infection and can bring serious difficulties for clinical treatment of *L. hongkongensis* infection.

## Conclusion

The genome sequences of *L. hongkongensis* reported here is a valuable resource for further studies investigating the newly discovered foodborne pathogen. The comprehensive analysis of the genetic evolution, virulence factors, carbohydrate-active enzymes can provide a snapshot of the genetic and pathogenic characteristics of *L. hongkongensis*. Prediction of antimicrobial resistance gene and susceptibility analysis of clinical drugs gives high-quality reference information for drug choice, which will benefit the environmental and clinical control of *L. hongkongensis*.

## Data Availability Statement

The datasets presented in this study can be found in online repositories. The names of the repository/repositories and accession number(s) can be found in the article/Supplementary Material.

## Ethics Statement

The animal study was reviewed and approved by the Medical Ethics Committee of Zhujiang Hospital, Southern Medical University.

## Author Contributions

P-BY performed the data analyses and wrote the manuscript. YZ contributed significantly to analysis and manuscript preparation. J-HZ and J-HL performed the experiment. E-ZC, W-TL, L-JW, and Y-XZ helped perform the sample collection, bacteria isolation, data analysis, and constructive discussions. D-QC conceived and designed the study and co-wrote the manuscript. All authors contributed to the article and approved the submitted version.

## Conflict of Interest

The authors declare that the research was conducted in the absence of any commercial or financial relationships that could be construed as a potential conflict of interest.

## Publisher’s Note

All claims expressed in this article are solely those of the authors and do not necessarily represent those of their affiliated organizations, or those of the publisher, the editors and the reviewers. Any product that may be evaluated in this article, or claim that may be made by its manufacturer, is not guaranteed or endorsed by the publisher.

## Footnotes

1
https://www.bioinformatics.babraham.ac.uk/projects/fastqc/

2
https://www.ncbi.nlm.nih.gov/

## Supplementary Material

Supplementary Table 1 The general characteristics of *L. hongkongensis genomes*.

Supplementary Table 2 The gene presence and absence matrix and annotation of allidentified genes.

Supplementary Table 3 The functions of core genes were characterized by matchingthe sequences with the COG database.

Supplementary Table 4 Virulence factors detected in the 49 *L. hongkongensis* strains.

Supplementary Table 5 CAZyme profiling of the 49 *L. hongkongensis* strains.

Supplementary Table 6 Antibiotic resistance genes predicted and antimicrobial susceptibility for drugs of *L. hongkongensis* strains.
